# The Effect of Neoadjuvant Treatment on the Number of Lymph Node Dissection and Prognosis in Locally Advanced Rectal Cancer and Other Factors Affecting Lymph Node Metastasis

**DOI:** 10.7759/cureus.95368

**Published:** 2025-10-25

**Authors:** Kubilay Kenan Ozluk, Niyazi Karaman

**Affiliations:** 1 Department of Surgical Oncology, Gülhane Training and Research Hospital, Ankara, TUR; 2 Department of General Surgery, Ankara Oncology Training and Research Hospital, Ankara, TUR

**Keywords:** lymph node, metastasis, neoadjuvant, pathology, rectal cancer

## Abstract

Introduction

The current standard treatment for locally advanced rectal cancer is neoadjuvant chemoradiotherapy (CRT), followed by total mesorectal excision. The removal of at least 12 lymph nodes is recommended to accurately stage the disease and predict prognosis, but surgery following neoadjuvant therapy often fails to reach all 12 lymph nodes. This study investigated the changes in lymph node count following chemoradiotherapy and the factors contributing to this change. We also examined the impact of these factors on prognosis.

Materials and methods

A retrospective review was conducted on 250 patients diagnosed with locally advanced rectal cancer between 2012 and 2019. These patients were divided into two groups: those who received neoadjuvant chemoradiotherapy (NACRT) and those who did not. The number of lymph nodes between the two groups and the factors affecting this number were investigated. The distribution of variables was measured using the Kolmogorov-Smirnov test. Cox regression (univariate-multivariate) and Kaplan-Meier regression were used for survival analysis.

Results

Of the patients, 135 received neoadjuvant chemoradiotherapy, and 115 began treatment with initial surgery. The number of lymph nodes was statistically significantly lower in the chemoradiotherapy group (9.5 versus 15.1, p=0.000). Patient comorbidities influenced the initial treatment option and directly affected the number of lymph nodes. Furthermore, tumor location, the use of total mesorectal excision, and the pathological response of the tumor to chemoradiotherapy significantly affected the number of lymph nodes. Multivariate analysis revealed that circumferential resection margin (CRM) negativity was associated with disease-free survival (DFS), while overall survival (OS) was associated with metastatic lymph node status (p=0.01), the lack of neoadjuvant therapy (p=0.00), and impaired total mesorectal excision integrity (p=0.01).

Conclusion

Chemoradiotherapy is the first-line treatment for locally advanced rectal cancer. Chemoradiotherapy reduces the number of lymph nodes. We advocate that pathological tumor response, in addition to lymph node count, should be considered to predict patient prognosis.

## Introduction

Rectal cancer is one of the leading causes of cancer-related deaths worldwide. At the time of diagnosis, 75% of the patients do not have distant organ metastases. Lymph node involvement is one of the most important prognostic factors in patients with colorectal cancer. Tumor, node, and metastasis (TNM) classification is used for staging in rectal cancer. The patients with T3-4 N0 disease or any T stage with N+ involvement are considered to have locally advanced disease. The primary treatment for locally advanced rectal cancer is regional radiotherapy (RT) combined with systemic chemotherapy (CT). In systemic chemotherapy, agents such as 5-fluorouracil, capecitabine, oxaliplatin, and irinotecan are used, while both short-course and long-course radiotherapy options are available. In surgical treatment, total mesocolic excision (TME) is the gold standard.

Neoadjuvant therapy is a treatment method for locally advanced rectal cancer that aims to preserve the sphincter, prevent local-regional recurrence, and improve survival [1]. While this approach is clearly effective in preventing local recurrence, data on its effectiveness in preventing distant metastasis are not yet conclusive. Therefore, the addition of various systemic chemotherapy agents to neoadjuvant therapy remains a subject of ongoing research. In general, long-course chemoradiotherapy (CRT) protocols are preferred for patients in whom tumor downstaging is desired, whereas short-course radiotherapy protocols are preferred when the primary goal is to prevent local-regional recurrence.

In patients with colorectal cancer, it is recommended that at least 12 lymph nodes be removed for accurate staging and prognosis. High serum carcinoembryonic antigen (CEA) levels, lymphovascular invasion, large tumor diameter, and low levels of MSH6 and MSH1 are risk factors for lymph node metastasis [2]. The number and ratio of lymph node metastases are also directly associated with poor survival. Therefore, accurately staging the lymph node status of the disease is highly important. However, in patients receiving neoadjuvant therapy, the number of lymph nodes often does not reach 12. Regional radiotherapy reduces the average number of lymph nodes by two, while the addition of chemotherapy to radiotherapy reduces the number by an average of four. Therefore, the removal of 12 lymph nodes as a cut-off value may be questionable in patients with rectal cancer receiving neoadjuvant therapy [3-5].

This study aimed to investigate how neoadjuvant chemoradiotherapy (NACRT) alters the number of lymph nodes in rectal cancer and how this affects disease-free survival (DFS) and overall survival (OS). It was planned to contribute new findings to the literature by examining the relationship between the number of lymph nodes removed and tumor location, the chemotherapy protocol administered concurrently with radiotherapy, the waiting period after chemoradiotherapy, the circumferential resection margin (CRM) and TME, the pathological response type, the type of surgery, age, gender, and body mass index (BMI).

## Materials and methods

Study population

The sample size of this study was calculated as a total of 196 patients, with α=0.05, power (1-β)=0.95, a case-to-intervention group ratio of 1:1, a medium effect size, and a one-sided effect size of at least 98 for each group, using the G*Power program (Heinrich-Heine-Universität Düsseldorf, Düsseldorf, Germany) [6].

This study was conducted through a retrospective review of patients who were diagnosed with rectal cancer between January 2012 and December 2019 at the departments of general surgery and surgical oncology of the University of Health Sciences, Ankara Oncology Training and Research Hospital. It included patients who received neoadjuvant therapy, followed by surgery, as well as those who underwent primary surgery without receiving neoadjuvant therapy.

Patients who did not have rectal cancer, those whose surgeries were performed at external centers, those who received adjuvant or neoadjuvant therapy at external centers, patients under 18 years of age, patients who underwent intervention for benign reasons, patients considered inoperable, pregnant patients, emergency cases, patients who developed cancer on the background of inflammatory bowel disease, and those with metachronous or synchronous colon tumors were excluded from the study. Patients who did not have liver metastases at the time of diagnosis but had radiologically undetectable liver metastases after neoadjuvant treatment and underwent curative surgery were included in the study.

Study design

Approval for the study was obtained from the Medical Specialization and Education Board Ethics Committee of Ankara Oncology Hospital under decision number 2020-12/919. A total of 250 patients diagnosed with rectal cancer and meeting the criteria between January 2012 and December 2019 in the surgical and surgical oncology departments of Ankara Oncology Hospital were identified. These patients were divided into two main groups: those who received neoadjuvant therapy and those who did not. The following variables were recorded for these patients: age, sex, body mass index (BMI), T status, the number of dissected lymph nodes, N status, stage, the number of positive lymph nodes, tumor location, whether radiotherapy (RT) was received, chemotherapy (CT) protocols administered, waiting period after treatment, circumferential resection margin (CRM) status, whether TME was performed, pathological tumor response to neoadjuvant therapy (tumor regression score based on modified Ryan scoring) (tumor regression grade {TRG}), the type of surgery performed, the presence of recurrence, the duration of follow-up, and American Society of Anesthesiologists (ASA) score. Patients were grouped based on the number of dissected lymph nodes as ≤10 and >10. The number of metastatic and total lymph nodes in the specimen was recorded. Tumor location was classified based on the distance from the anal verge as lower (0-4 cm), middle (5-8 cm), and upper rectum (9-12 cm). Patients who received adjuvant radiotherapy and systemic therapy were also recorded. The types of surgeries performed were classified as low anterior resection (LAR), abdominoperineal resection (APR), laparoscopic LAR, and laparoscopic APR.

The resected surgical specimen was examined using standard methods. First, the pericolic adipose tissue was separated from the specimen and kept in formalin, and the lymph nodes were palpated and carefully dissected. If fewer than 12 lymph nodes were found, the specimen was re-examined. Paraffin blocks were stained with hematoxylin and eosin.

In patients who received neoadjuvant therapy, the pathological response of the tumor was recorded according to the modified Ryan scoring system: grade 0 (complete pathological response), grade I (near-complete response), grade II (minimal response), and grade III (poor response).

Patients with no tumor seen at the radial surgical margin were considered CRM-negative.

For postoperative follow-up, patients were called in for checkups every three months during the first two years, where physical examination and tumor markers were evaluated. Whole-body scans were performed with thoracic and abdominal computed tomography every six months, and annual colonoscopic evaluations were conducted.

Statistical analysis

Data were analyzed by using SPSS version 22.00 (IBM Corp., Armonk, NY). The Kolmogorov-Smirnov and Levene tests were used to assess the normality and homogeneity of scaled data. Pearson's chi-square and Fisher's exact tests were used to evaluate the nominal data of each group, Student's t-test for parametric scaled data, and the Mann-Whitney U test for nonparametric scaled data. Univariate and multivariate hazard ratios (HRs) were calculated using the Cox regression model. The results of the regression analysis were expressed as hazard ratios (HRs) and 95% confidence intervals (CIs). A p-value of <0.05 was considered statistically significant.

## Results

Patient characteristics

A total of 250 patients diagnosed with rectal cancer and operated on between January 2012 and December 2019 at the General Surgery and Surgical Oncology Clinics of Ankara Oncology Hospital were included in the study. The median age of the patients was 67 years (range: 28-96). Of the patients, 153 (61.2%) were men. Of the operated patients, 96.8% underwent surgery with an ASA II-III score. The average number of lymph nodes dissected from the pathology specimens was 12.1 (range: 0-40). In 238 patients (95.2%), the number of lymph nodes was found to be ≤10. When the new pathological stages after neoadjuvant treatment were evaluated, 63.2% of the patients were found to be in stages II-III. In 18 patients (7.2%) within the study group, liver metastasis that could not be detected on preoperative imaging was identified, and curative metastasectomy was performed. The distribution of tumor localization (lower-middle-upper) was homogeneous across the entire group.

Neoadjuvant treatment was administered to 135 patients (54%). Almost all of the patients received long-course radiotherapy, often in combination with capecitabine and 5-fluorouracil during the waiting period. In the group that underwent surgery first, adjuvant folinic acid, fluorouracil, and oxaliplatin (FOLFOX) use was more prominent. In the neoadjuvant treatment group, 116 patients (85.9%) received 5-fluorouracil+capecitabine, while 19 patients received FOLFOX.

In 227 patients (90.8%), CRM was found to be negative. Among the 135 patients who received neoadjuvant treatment, a complete or near-complete pathological response was achieved in 48 patients (35.6%). The average waiting time after neoadjuvant treatment was determined to be 8.5 (±4.9) weeks. During an approximately 2.5-year follow-up period, local recurrence was observed in 18 patients (7.2%), and mortality was observed in 98 patients (39.2%). The demographic and clinical characteristics of the patients are summarized in Table 1.

**Table 1:** Demographic and clinical characteristics of the patients BMI, body mass index; ASA, American Society of Anesthesiologists; FOLFOX, folinic acid, fluorouracil, and oxaliplatin; CRM, circumferential resection margin; TME, total mesocolic excision; LAR, low anterior resection; APR, abdominoperineal resection

			Minimum-Maximum			Median	Mean (%)±SD/n (%)		
Age			28.0	-	96.0	67.0	66.4	±	12.2
Sex		Female					97		(38.8%)
		Male					153		(61.2%)
BMI (kg/m^2^)			21.0	-	35.0	27.0	27.1	±	2.5
ASA score		I					4		(1.6%)
		II					143		(57.2%)
		III					99		(39.6%)
		IV					4		(1.6%)
Number of lymph nodes			0.0	-	40.0	11.5	12.1	±	7.4
Lymph node		≤10					238		(95.2%)
		>10					12		(4.8%)
Positive lymph node			0.0	-	24.0	0.0	1.7	±	3.5
positive lymph node rate			0.0	-	1.0	0.0	0.1	±	0.2
Stage		0					16		(6.4%)
		I					58		(23.2%)
		II					74		(29.6%)
		III					84		(33.6%)
		IV					18		(7.2%)
T stage		0					18		(7.2%)
		I					15		(6.0%)
		II					56		(22.4%)
		III					124		(49.6%)
		IV					37		(14.8%)
N stage		0					154		(61.6%)
		I					56		(22.4%)
		II					39		(15.6%)
		III					1		(0.4%)
Tumor location		Inferior					88		(35.2%)
		Medium					86		(34.4%)
		Superior					76		(30.4%)
Radiotherapy	Neoadjuvant						135		(54.0%)
	Adjuvant						58		(23.2%)
	No adjuvant						57		(22.8%)
Chemotherapy	5-Fluorouracil+capecitabine						151		(60.4%)
	FOLFOX						59		(23.6%)
	(-)						40		(16.0%)
CRM	(-)						227		(90.8%)
	(+)						23		(9.2%)
TME	(+)						238		(95.2%)
	(-)						12		(4.8%)
Pathological response	0						21		(15.6%)
	I						27		(20.0%)
	II						55		(41.5%)
	III						32		(23.7%)
Type of surgery									
LAR							104		(41.6%)
Laparoscopic LAR							57		(22.8%)
APR							57		(22.8%)
Laparoscopic APR							30		(12.0%)
Total colectomy							2		(0.8%)
Neoadjuvant	Present						115		(46.0%)
	Absent						135		(54.0%)
Treatment waiting time (weeks)			1.0	-	52.0	8.0	8.5	±	4.9
Relapse	(-)						232		(92.8%)
	(+)						18		(7.2%)
Mortality	(-)						152		(60.8%)
	(+)						98		(39.2%)
Follow-up period (weeks)			1.0	-	100.0	24.0	27.9	±	24.1

Comparison of neoadjuvant groups with clinicopathological parameters

The mean age of the patients in the group receiving neoadjuvant therapy was found to be statistically significantly lower compared to the group not receiving neoadjuvant therapy (64.7% versus 68.4%, p=0.022). This was thought to be due to younger patients tolerating neoadjuvant therapy better and therefore starting treatment with primarily neoadjuvant chemoradiotherapy, whereas it was thought that older patients began treatment with surgery due to their inability to tolerate chemotherapy. In the group receiving neoadjuvant therapy, the total number of lymph nodes was significantly lower compared to the group not receiving neoadjuvant therapy (9.5 versus 15.1, p=0.000). When the number of lymph node harvesting was considered, the number of patients with 10 or fewer lymph nodes removed was higher in the neoadjuvant therapy group (61.5% versus 25.2%, p=0.000). In other words, the majority of patients with 10 or fewer lymph nodes in the specimen were in the group that received neoadjuvant therapy. The number and rate of positive lymph nodes were also significantly lower in the neoadjuvant therapy group (p<0.05). As a result of this, when considering the new pathological stages, the rate of stage II-III disease in the group receiving neoadjuvant CRT was 52.6%, while this rate was 75.6% in the other group, mostly in favor of stage III; this difference was found to be statistically significant (p<0.001) (Table 2).

**Table 2:** Statistical evaluation of patient groups that received and did not receive neoadjuvant therapy Statistically significant values are shown in bold text ^m^Mann-Whitney U test ^X²^Ki-kare test (Fisher's test) BMI, body mass index; ASA, American Society of Anesthesiologists; FOLFOX, folinic acid, fluorouracil, and oxaliplatin; CRM, circumferential resection margin; TME, total mesocolic excision; LAR, low anterior resection; APR, abdominoperineal resection

			Neoadjuvant (-)					Neoadjuvant (+)						P		
			Mean±SD/n (%)			Median		Mean±SD/n (%)					Median			
Age			68.4	±	11.9	71.0		64.7		±	12.3		66.0	0.022		^m^
Sex	Female		45		(39.1%)			52			(38.5%)			0.921		^X²^
	Male		70		(60.9%)			83			(61.5%)					
BMI (kg/m^2^)			27.0	±	2.6	27.0		27.1		±	2.5		28.0	0.583		^m^
ASA score	I		2		(1.7%)			2			(1.5%)			0.873		^X²^
	II		65		(56.5%)			78			(57.8%)					
	III		46		(40.0%)			53			(39.3%)					
	IV		2		(1.7%)			2			(1.5%)					
Number of lymph nodes			15.1	±	7.8	14.0		9.5		±	5.9		9.0	<0.001		^m^
Lymph node	≤10		29		(25.2%)			83			(61.5%)			<0.001		^X²^
	>10		86		(74.8%)			52			(38.5%)					
Positive lymph node	-		62		(53.9%)			95			(70.4%)			0.007		^X²^
	+		53		(46.1%)			40			(29.6%)					
Positive lymph node			2.3	±	4.2	0.0		1.1		±	2.7		0.0	0.004		^m^
Positive lymph node rate			16.6%	±	(27.7%)	0.0%		10.1%		10.1±	(20.9%)		0.0%	0.018		^m^
New pathological stage	0		0		(0%)			16			(11.9%)			<0.001		^X²^
	I		24		(20.9%)			34			(25.2%)					
	II		38		(33.0%)			36			(26.7%)					
	III		49		(42.6%)			35			(25.9%)					
	IV		4		(3.5%)			14			(10.4%)					
T stage	0		0		(0%)			18			(13.3%)			<0.001		^X²^
	I		5		(4.3%)			10			(7.4%)					
	II		26		(22.6%)			30			(22.2%)					
	III		60		(52.2%)			64			(47.4%)					
	IV		24		(20.9%)			13			(9.6%)					
N stage	0		62		(53.9%)			92			(68.1%)			0.460		^X²^
	I		26		(22.6%)			30			(22.2%)					
	II		26		(22.6%)			13			(9.6%)					
	III		1		(0.9%)			0			(0%)					
Tumor location	Inferior		23		(20%)			65			(48.1%)			<0.001		^X²^
	Medium		34		(29.6%)			52			(38.5%)					
	Superior		58		(50.4%)			18			(13.3%)					
Radiotherapy+chemotherapy		Capecitabine		40		(34.8%)			116			(85.9%)		<0.001	^X²^	
		FOLFOX		40		(34.8%)			19			(14.1%)				
		None		35		(30.4%)										
CRM		(-)		102		(88.7%)			125			(92.6%)		0.288	^X²^	
		(+)		13		(11.3%)			10			(7.4%)				
TME		(+)		112		(97.4%)			126			(93.3%)		0.135	^X²^	
		(-)		3		(2.6%)			9			(6.7%)				
Type of surgery																
LAR				63		(54.8%)			41			(30.4%)		<0.001	^X²^	
Laparoscopic LAR				28		(24.3%)			29			(21.%)		0.590	^X²^	
APR				14		(12.2%)			43			(31.9%)		<0.001	^X²^	
Laparoscopic APR				9		(7.8%)			21			(15.6%)		0.610	^X²^	
Total colectomy				1		(0.9%)			1			(0.7%)		1.000	^X²^	
Relapse		(-)		103		(89.6%)			129			(95.6%)		0.068	^X²^	
		(+)		12		(0.4%)			6			(4.4%)				
Mortality		(-)		67		(58.3%)			85			(63.0%)		0.448	^X²^	
		(+)		48		(41.7%)			50			(37.0%)				
Follow-up period (weeks)				30.0	±	25.5	24.0		26.1		±	22.8	21.0	0.279	^m^	

Comparison of lymph node dissection groups with clinicopathological parameters

When patients were divided into two groups based on the cut-off value of 10 for the number of lymph nodes retrieved (group with 10 or fewer and group with more than 10), the group with more than 10 lymph nodes removed was found to have a significantly higher number and rate of positive lymph nodes and more advanced new pathological stage. Additionally, tumor location (more frequently located in the middle and upper rectum), higher rate of TME performed, poor pathological response, and higher recurrence rate were also more common in the group with more than 10 lymph nodes removed. If we consider this situation in terms of cause and effect, the number of lymph nodes is higher in the group that did not receive neoadjuvant therapy. Naturally, in this group, the higher rate of positive lymph nodes and the more advanced pathological stage compared to the neoadjuvant-treated group are expected findings.

The higher rate of local recurrence in the group with more than 10 lymph nodes removed can be explained by the effect of neoadjuvant therapy, as neoadjuvant therapy reduces both the number of lymph nodes and the rate of local recurrence. The fact that patients with poor pathological response were significantly more frequent in the group with a higher number of lymph nodes is again associated with their lower response to neoadjuvant therapy.

The number of lymph nodes was higher in the group that underwent TME because the rectal mesentery was fully excised and no residual tissue was left, and this was found to be statistically significant. Since the rate of neoadjuvant therapy is higher in lower rectal cancer compared to middle and upper rectal cancer, lower rectal cancer was statistically significantly more frequent in the group with fewer lymph nodes removed (Table 3).

**Table 3:** Comparison of groups according to the number of lymph nodes Statistically significant values are shown in bold text ^m^Mann-Whitney U test ^X²^Ki-kare test (Fisher's test) BMI, body mass index; ASA, American Society of Anesthesiologists; FOLFOX, folinic acid, fluorouracil, and oxaliplatin; CRM, circumferential resection margin; TME, total mesocolic excision; LAR, low anterior resection; APR, abdominoperineal resection; RT, radiotherapy; CT, chemotherapy

		Number of Lymph Nodes ≤10					Number of Lymph Nodes >10				P	
		Mean±SD/n, (%)		Median			Mean±SD/n, (%)			Median		
Age		67.8	±	12.5	68.0		65.2	±	11.9	66.0	0.103	^m^
	Female	42		(37.5%)			55		(39.9%)		0.704	^X²^
	Male	70		(62.5%)			83		(60.1%)			
BMI (kg/m^2^)		26.7	±	2.5	27.0		27.4	±	2.5	28.0	0.058	^m^
ASA score	I	2		(1.8%)			2		(1.4%)		0.042	^X²^
	II	56		(50.0%)			87		(63.0%)			
	III	52		(46.4%)			47		(34.1%)			
	IV	2		(1.8%)			2		(1.4%)			
Positive lymph node	(-)	83		(74.1%)			74		(53.6%)		0.001	^X²^
	(+)	29		(25.9%)			64		(46.4%)			
Positive lymph node		0.7	±	1.5	0.0		2.4	±	4.4	0.0	<0.001	^m^
Positive lymph node rate		12.6%	±	25.7%	0.0%		13.5%	±	23.5%	0.0%	0.026	^m^
New pathological stage	0	14		(12.5%)			2		(1.4%)		0.002	^X²^
	I	30		(26.8%)			28		(20.3%)			
	II	32		(28.6%)			42		(30.4%)			
	III	28		(25.0%)			56		(40.6%)			
	IV	8		(7.1%)			10		(7.2%)			
T stage	0	15		(13.4%)			3		(2.2%)		0.003	^X²^
	I	9		(8.0%)			6		(4.3%)			
	II	26		(23.2%)			30		(21.7%)			
	III	51		(45.5%)			73		(52.9%)			
	IV	11		(9.8%)			26		(18.8%)			
N stage	0	81		(72.3%)			73		(52.9%)		0.125	^X²^
	I	18		(16.1%)			38		(27.5%)			
	II	13		(11.6%)			26		(18.8%)			
	III	0		(0.0%)			1		(0.7%)			
Tumor location	Inferior	55		(49.1%)			33		(23.9%)		<0.001	^X²^
	Medium	31		(27.7%)			55		(39.9%)			
	Superior	26		(23.2%)			50		(36.2%)			
RT+CT	5-Fluorouracil+capecitabine	83		(74.1%)			68		(49.3%)		<0.001	^X²^
	FOLFOX	15		(13.4%)			44		(31.9%)			
	(-)	14		(12.5%)			26		(18.8%)			
CRM	(-)	105		(93.8%)			122		(88.4%)		0.146	^X²^
	(+)	7		(6.3%)			16		(11.6%)			
TME	(+)	102		(91.1%)			136		(98.6%)		0.006	^X²^
	(-)	10		(8.9%)			2		(1.4%)			
Pathological response	0	17		(20.5%)			4		(7.7%)		0.014	^X²^
	I	18		(21.7%)			9		(17.3%)			
	II	36		(43.4%)			20		(38.5%)			
	III	12		(14.5%)			19		(36.5%)			
Type of surgery												
LAR		40		(35.7%)			64		(46.4%)		0.089	^X²^
Laparoscopic LAR		17		(15.2%)			40		(29.0%)		0.010	^X²^
APR		35		(1.3%)			22		(15.9%)		0.004	^X²^
Laparoscopic APR		19		(17.0%)			11		(8.0%)		0.030	^X²^
Total colectomy		1		(0.9%)			1		(0.7%)		1.000	^X²^
Neoadjuvant	(-)	29		(25.9%)			86		(62.3%)		<0.001	^X²^
	(+)	83		(74.1%)			52		(37.7%)			
Treatment waiting time		8.7	±	3.0	8.0		8.3	±	7.0	8.0	0.064	^m^
Relapse	(-)	108		(96.4%)			124		(89.9%)		0.046	^X²^
	(+)	4		(3.6%)			14		(10.1%)			
Mortality	(-)	63		(56.3%)			89		(64.5%)		0.184	^X²^
	(+)	49		(43.8%)			49		(35.5%)			
Follow-up period (weeks)		23.4	±	23.0	17.5		31.6	±	24.4	26.0	0.003	^m^

The association between clinicopathological factors and disease-free survival (DFS) and overall survival (OS)

In the univariate analysis, factors affecting disease-free survival duration were identified as lower new pathological stage (p=0.02), negative CRM (p=0.00), the presence of TME integrity (p=0.04), the presence of complete or near-complete pathological response (p=0.02), the higher number of harvested lymph nodes, and the lower number of pathologically positive lymph nodes (p=0.01). In the multivariate analysis, only CRM negativity was found to be associated with disease-free survival (p=0.00). Factors affecting disease-free survival are summarized in Table 4.

**Table 4:** Factors affecting disease-free survival Statistically significant values are shown in bold text BMI, body mass index; ASA, American Society of Anesthesiologists; CRM, circumferential resection margin; TME, total mesocolic excision; LN, lymph node; HR, hazard ratio; CI, confidence interval; LR, likelihood ratio

	Disease-Free Survival									
	Univariate model					Multivariate model				
	HR	95% CI			P	HR	95% CI			P
Age	0.99	0.95	-	1.02	0.454					
BMI	1.08	0.89	-	1.31	0.427					
Sex	1.01	0.39	-	2.63	0.984					
ASA	1.00	0.42	-	2.38	0.996					
T stage	3.27	1.59	-	6.73	0.001					
N stage	1.90	1.09	-	3.32	0.023					
Tumor location	0.93	0.52	-	1.68	0.817					
CRM	10.12	3.79	-	27.3	<0.001	34.27	6.16	-	190.74	<0.001
TME	4.59	1.05	-	20.09	0.043					
Pathological response	5.76	1.30	-	25.41	0.021					
Type of surgery	1.22	0.81	-	1.83	0.351					
Number of LN >10	2.12	0.70	-	6.44	0.186					
Number of positive LN	1.11	1.02	-	1.20	0.018					
Treatment waiting time	0.96	0.72	-	1.30	0.812					
Cox regression (forward LR)										

In the univariate analysis, unfavorable factors associated with overall survival were older age (p=0.00), higher ASA score (p=0.00), higher new pathological stage (p=0.00), the absence of neoadjuvant therapy (p=0.00), positive CRM (p=0.00), the disruption of TME integrity (p=0.00), lower pathological response rate (p=0.00), the higher number and ratio of positive lymph nodes (p=0.00), and early surgery without an adequate waiting period after CRT. In the multivariate analysis, the factors associated with shorter survival were found to be advanced N stage (p=0.01), the absence of CRT (p=0.00), and the disruption of TME integrity (p=0.01). Factors affecting overall survival are summarized in Table 5.

**Table 5:** Factors affecting overall survival Statistically significant values are shown in bold text BMI, body mass index; ASA, American Society of Anesthesiologists; CRM, circumferential resection margin; TME, total mesocolic excision; LN, lymph node; HR, hazard ratio; CI, confidence interval; CRT, chemoradiotherapy; LR, likelihood ratio

	Overall Survival									
	Univariate model					Multivariate model				
	HR	95% CI			P	HR	95% CI			P
Age	1.04	1.02	-	1.06	<0.001					
BMI	0.99	0.91	-	1.07	0.738					
Sex	1.14	0.75	-	1.72	0.544					
ASA	1.67	1.17	-	2.37	0.004					
New stage	1.47	1.20	-	1.80	<0.001					
T stage	1.59	1.25	-	2.02	<0.001					
N stage	1.70	1.35	-	2.15	<0.001	1.58	1.09	-	2.28	0.016
Tumor location	0.86	0.66	-	1.10	0.231					
Neoadjuvant treatment	1.44	1.12	-	1.85	0.005	2.45	1.56	-	3.83	<0.001
CRM	2.89	1.74	-	4.78	<0.001					
TME	3.10	1.61	-	5.99	0.001	2.87	1.27	-	6.45	0.011
Pathological response	1.58	1.16	-	2.17	0.004					
Type of surgery	1.04	0.87	-	1.25	0.645					
Number of LN >10	0.98	0.96	-	1.01	0.216					
Number of LN >10	0.65	0.44	-	0.97	0.033					
Number of positive LN	1.08	1.04	-	1.13	<0.001					
Positive LN rate	4.33	2.30	-	8.16	<0.001					
LN with CRT effect	1.19	0.64	-	2.21	0.577					
Treatment waiting time	0.87	0.79	-	0.96	0.007					
Relapse	1.61	0.91	-	2.85	0.099					
Cox regression (forward LR)										

## Discussion

Lymph node involvement is one of the most important prognostic indicators in colorectal cancers [4]. The total number of lymph nodes removed is associated with survival, and at least 12 lymph nodes should be excised to ensure accurate staging [4-9]. There are studies showing that the number of lymph nodes decreases in patients who receive neoadjuvant therapy [10-14]. Even with optimal dissection, it may not be possible to remove 12 lymph nodes in patients receiving neoadjuvant treatment. Neoadjuvant therapy leads to fibrosis, lymphocyte depletion, and atrophy in lymph nodes, thereby reducing their count [15-19]. In light of this evidence, lymph node count alone is not sufficient to predict prognosis in patients who have undergone neoadjuvant therapy. Additional factors must be analyzed alongside this parameter. One of the aims of our study was to contribute to the literature by revealing these factors.

In this study, consistent with previous literature, the average number of lymph nodes removed in the group that did not receive neoadjuvant therapy was 15.1, while in the group that received neoadjuvant therapy, this number was found to be 9.5. When fewer than 12 lymph nodes are removed, the staging may be considered insufficient, causing physicians to feel uncertain about prognosis prediction. However, studies indicate that in patients receiving neoadjuvant therapy, the lymph node ratio and the logarithmic value of this ratio are more meaningful predictors of prognosis than the absolute lymph node count [20]. As the metastatic lymph node ratio increases, survival time decreases [21]. This study also demonstrated that neoadjuvant therapy reduces the number and ratio of metastatic lymph nodes, lowers the new pathological stage, and improves overall survival. Nevertheless, there remains debate in the literature regarding the appropriate cut-off value for the lymph node ratio [22-28].

Another important prognostic variable in patients undergoing TME after neoadjuvant therapy is the pathological tumor response rate. In rectal cancer, the response to neoadjuvant treatment is evaluated by the tumor regression grade (TRG). According to this system, grades 0-1 indicate a good response, while grades 2-3 indicate a poor response. Numerous studies in the literature have shown that patients with good tumor regression scores also have lower numbers of lymph nodes removed [29-31]. For patients receiving neoadjuvant therapy, some studies suggest that removing 7-9 lymph nodes may be sufficient for standard staging, rather than the recommended 12 nodes [14,32-34]. In a meta-analysis by Petrelli et al., pathological complete response was shown to be a better prognostic indicator for survival than lymph node count [35].

In current practice, it is recommended to remove the maximum number of lymph nodes possible. However, it should not be forgotten that for determining prognosis, not only the lymph node count alone but also the lymph node count together with the pathological response rate is important. In the study conducted by Narayanan et al., the best survival was observed in patients who achieved pathological complete response and had more than 12 lymph nodes removed. This group was followed by patients with pathological complete response but fewer than 12 lymph nodes removed and then by patients who underwent surgery without neoadjuvant therapy and had more than 12 lymph nodes removed. The worst survival outcomes were seen in patients with locally advanced disease who underwent primary surgery and had fewer than 12 lymph nodes removed [17]. Viewed from a different perspective, in patients receiving NACRT, detecting fewer than 12 lymph nodes in the specimen may also indicate a good pathological tumor response. Indeed, in the study by Kim et al., a low lymph node count was shown to be associated with tumor response and positively correlated with prognosis [36]. In this study as well, patients with a good pathological response to neoadjuvant therapy had significantly lower lymph node counts, and survival in this group was longer compared to patients with a poor response.

Extending the waiting period after CRT applications is thought to increase the rate of pathological tumor response [37]. In the study by Terzi et al. comparing eight with 12 weeks of waiting after NACRT, no difference was found in disease-free survival and overall survival [38]. In this study, the mean waiting time after neoadjuvant treatment was 8.5 weeks (1-52). We think that the COVID-19 pandemic also has an effect on the prolongation of the waiting period. When evaluated in terms of lymph node count and local-regional recurrence, no effect of the extended waiting period was observed. However, the effect of waiting time on mortality, which was revealed in univariate analysis, could not be demonstrated in multivariate analysis.

Limitations of the study

The most significant limitation of our study is that it was conducted as a retrospective evaluation of a patient series. Additionally, pelvic MRI, which should be routinely performed as a standard in preoperative staging, was not applied routinely to all our patients. Instead, computed tomography (CT) was used more frequently for systemic metastasis screening and local staging. This choice was influenced by the long waiting times for MRI appointments and the surgical team's greater expertise in interpreting CT scans. Due to the more frequent use of CT, the T stage was prioritized over the N stage when selecting patients for neoadjuvant treatment, which has also affected our study results.

In the study group, open surgery was performed more frequently. While open surgeries were conducted by a broader surgical team, laparoscopic procedures were performed by a single team. Additionally, there was heterogeneity among the operated patients due to differences in oncological surgical approaches among the surgeons.

The pathological evaluation of specimens was not performed by a single team, and the chemotherapy regimen was not given by a single team.

## Conclusions

We found that the number of dissected lymph nodes was lower in the patients who received neoadjuvant therapy compared to those who did not. We determined that this difference was influenced by the pathological response, the fact that younger patients received neoadjuvant therapy more frequently, the distal location of rectal tumors, the effectiveness of the applied NACRT, and whether TME was performed. The factors affecting local-regional recurrence were identified as CRM positivity, the number of metastatic lymph nodes and their ratio to the total number of lymph nodes, new advanced pathological stage, the lack of TME integrity, and poor pathological response rate. The factors affecting mortality were identified as not receiving NACRT, advanced patient age, high ASA score, high metastatic lymph node ratio, advanced new clinical stage, positive CRM, incomplete TME, low pathological response rate to CRT, and short waiting period after CRT.

In conclusion, lymph node status is highly important in terms of survival in rectal cancer. However, in patients receiving neoadjuvant therapy, it may not be possible to reach 12 lymph nodes for staging and prognosis. We are of the opinion that evaluating the pathological response to neoadjuvant therapy together with lymph node status would be more meaningful in terms of survival.
